# Effectiveness of an intervention designed based on the Health Action Process Approach on obesity surgery outcomes in patients who have undergone bariatric surgery after one year: A randomized controlled trial

**DOI:** 10.1371/journal.pone.0314316

**Published:** 2025-04-09

**Authors:** Maryam Maghsoodlo, Elham Shakibazadeh, Mehdi Yaseri, Zeinab Mokhtari, Maryam Barzin, Yahya Salimi

**Affiliations:** 1 Health Management and Social Development Research Center, Golestan University of Medical Sciences, Gorgan, Iran; 2 Department of Health Education and Promotion, School of Public Health, Tehran University of Medical Sciences, Tehran, Iran; 3 Department of Epidemiology and Biostatistics, Tehran University of Medical Sciences (TUMS), Tehran, Iran; 4 Nutrition and Food Security Research Center, Isfahan University of Medical Sciences, Isfahan, Iran; 5 Obesity Research Center, Research Institute for Endocrine Sciences, Shahid Beheshti University of Medical Sciences, Tehran, Iran; 6 Social Development & Health Promotion Research Center, Health Institute, Kermanshah University of Medical Sciences, Kermanshah, Iran; King's College Hospital NHS Trust: King's College Hospital NHS Foundation Trust, UNITED KINGDOM OF GREAT BRITAIN AND NORTHERN IRELAND

## Abstract

**Background:**

Bariatric surgery is effective in treating severe obesity. However, surgery alone, without additional behavior change management, may not lead to optimal long-term weight loss and maintenance. This study aimed to evaluate an intervention designed based on the Health Action Process Approach to improve outcomes of obesity surgery in patients who underwent bariatric surgery in Tehran, Iran.

**Methods:**

In this randomized controlled trial, a total of 100 patients who had undergone bariatric surgery after the past year were randomly assigned to two intervention (n = 50) and control (n = 50) groups. The intervention group received educational intervention for two months. Health action process approach (HAPA) constructs, the Bariatric Surgery Self-Management Behaviors Questionnaire (BSSQ), dietary recall, blood chemistry parameters, BMI, percentage of body weight loss, and the International Physical Activity Questionnaire (IPAC) were measured at baseline and four months after the intervention. To compare the changes between the two groups before and four months after the educational intervention, the interaction of group and time was analyzed using the generalized estimating equation (GEE). A p-value of less than 0.05 was considered statistically significant.

**Results:**

The educational intervention resulted in improvements in various aspects of self-efficacy, including task and coping self-efficacy constructs (P = 0.02), action planning (P < 0.01) and behavioral intention (P < 0.01) related to diet self-management. There were also statistically significant improvements in action planning (P = 0.02), risk perception (P = 0.01) and Recovery self- efficacy (P = 0.01) related to the self-management of physical activity. There were significant improvements in the iron blood test results (P =  0.01) among the patients.

**Conclusion:**

Our intervention, designed based on the Health Action Process Approach, led to improvements in dietary and physical activity outcomes among patients who underwent bariatric surgery.

**Trial Registration:** Iran Randomized Clinical Trials IRCT20230722058887N1.

## Introduction

Obesity is a significant public health challenge across the globe [1]. The rate is rapidly growing in both developing and developed countries [2]. In 2022, 2.5 billion adults (18 years and older) were overweight, off those, 890 million were living with obesity [3,4]. The prevalence of severe obesity has significantly increased in Africa and the Middle East [2,5].

Bariatric Surgery (BS) is known as an effective treatment method for individuals with morbid obesity [6]. Although bariatric surgery yields significant results, it is not sustainable for all patients. [7]. Research studies indicate that weight regain and recurrence of type 2 diabetes and other diseases may occur after the surgery [8–14].

Research studies indicate that bariatric surgery alone is often an insufficient treatment [9,15–19]. Factors such as sedentary lifestyle, and failure to adhere to healthy diet can cause weight gain following bariatric surgery [20,21]. It is important to understand the obstacles that affect weight loss after bariatric surgery and/or factors that cause weight regain after the surgery in order to minimize and control their effects [6,22,23].

Self-management following bariatric surgery and the need to provide post-surgery healthy lifestyle education is crucial [24,25]. Cognitive interventions aimed at promoting proper nutritional behaviors and physical activity are essential for self-management improvement [25,26].

Theory-based interventions can help understand which specific strategies and approaches are effective in engaging patients in self-management and why [27]. The Health Action Process Approach (HAPA) is an appropriate and effective model for implementing active patient interventions [28]. HAPA describes the factors that influence the adoption and maintenance of health behaviors [29]. According to HAPA, behavior change consists of two consecutive phases: (1) a motivational phase that includes risk perceptions, outcome expectancies, and task self-efficacy, leading to formation of a behavioral intention; and (2) a volitional phase that includes maintenance self-efficacy, recovery self-efficacy, action planning, and coping planning, all of which contribute to the actual health behavior [29–33]. A number of interventions have successfully used HAPA to increase fruit and vegetable consumption. HAPA has also been applied in the context of diabetes [34–36].

The present study aimed to evaluate an intervention designed based on the HAPA to improve outcomes of obesity surgery in patients who have undergone bariatric surgery.

## Methods

### Research Design and Participants

This randomized controlled trial was part of a Ph.D thesis. The protocol of the study was approved by the Iranian Registry of Clinical Trials (IRCT20230722058887N1). We used the CONSORT checklist when writing the paper (Supporting File 1).

### Ethics approval and consent to participate

The Ethics Committee of Tehran University of Medical Sciences (IR.TUMS.SPH.REC.1400.230) approved this study and the study was carried out by the Helsinki Declaration. Ethical considerations were taken into account during data collection and analysis. The confidentiality of the participants was protected, and no personal information could be identified in any publications arising from the study. Participants were informed that their participation is entirely voluntary.

Informed verbal consent was obtained from all participants. The respondents were not teenagers; therefore, no parental consent was required, and they participated in the study with their informed consent.

**Fig 1:**
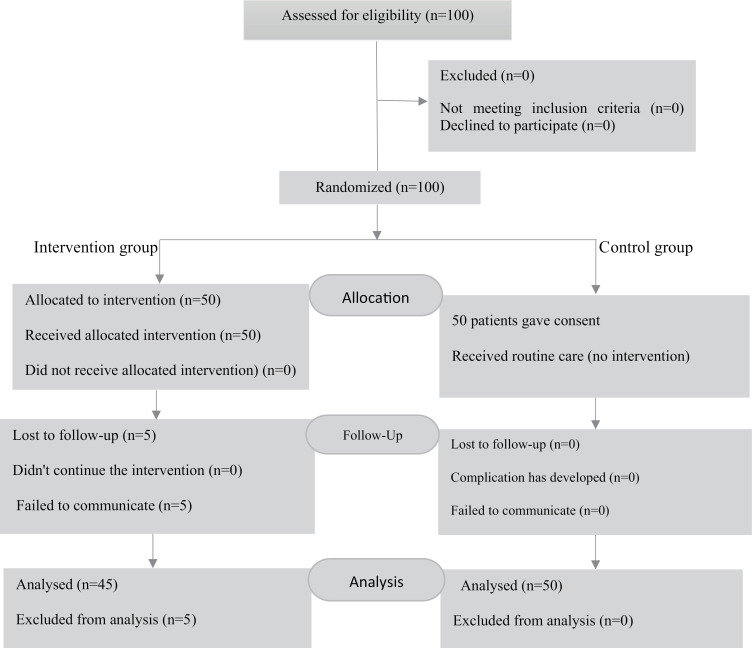
This flowchart illustrates the number of patients who entered the study at the outset, the number following the educational intervention, and the number four months after the intervention.

One hundred participants were selected from the obesity treatment cohort registry at Hakim Clinic in Tehran from April 2023 to November 2023. All participants were randomly assigned to two equal intervention and control groups. Study variables were measured at baseline and four months after the educational intervention. Fig 1 illustrates the participants at baseline in both the control and intervention groups, as well as their status following the intervention.

### Inclusion criteria

Patients who had undergone bariatric surgery at least one year ago and consented to participate in the study.

### Exclusion criteria

Patients who become pregnant during the study and the patients who did not participate in two thirds of the educational sessions.

### Sample size

Assuming an alpha error of 0.05 and a power of 80%, a total of 90 patients (n = 45 patients per group) were needed to detect an effect (a between-group difference of 6 scores of Bariatric Surgery Self-Management Behaviors Questionnaire change as this In our samples, in the pilot study, the score range was a maximum of 60 points, and we deemed 10% of it as indicative of significant changes.) with an assumed standard deviation of 10.08 (based on our pilot study involving 25 individuals). To account for a potential 10% drop in each group, 50 individuals were included in each group.

### Randomization

Participants were randomly assigned to two intervention and control groups using the permuted block randomization method. The randomly varying block length was set at 2, 4, and 6.

### Measures

We used a structured questionnaire based on HAPA, which consisted of two sections (Supplementary File 2). The first section focused on healthy diet and included seven constructs and 23 items. These constructs were: (1) task and coping self-efficacy, measured using a five-point Likert scale ranging from 1 (strongly disagree) to 5 (strongly agree) (3 items), (2) action planning (3 items), (3) coping planning (4 items), (4) recovery self-efficacy (3 items), (5) risk perception (4 items), (6) outcome expectancies (4 items), and (7) behavioral intention, measured using a five-point Likert scale ranging from 1 (strongly disagree) to 5 (strongly agree) (2 items). The second section, which focused on physical activity, consisted of seven constructs and 22 items. These included: (a) task and coping self-efficacy (3 items), (b) action planning (4 items), (c) coping planning (3 items), (d) recovery self-efficacy (3 items), (e) risk perception (3 items), (f) outcome expectancies (4 items), and (g) behavioral intention (2 items). These constructs were measured using five-interval Likert scales, ranging from 1 (strongly disagree) to 5 (strongly agree).

We also assessed self-management behaviors using the Bariatric Surgery Self-Management Behaviors Questionnaire (BSSQ). The BSSQ was designed and assessed by Welch et al. (2008). This questionnaire includes 32 items that assess seven behavioral domains: eating, drinking, protein consumption, physical activity, management of dumping syndrome, consumption of fruits, vegetables, and whole grains, and use of vitamin and mineral supplements [37]. Amini et al. assessed the validity and reliability of the questionnaire in Farsi language [38]. BSSQ items have a Likert scale format of “never,” “sometimes,” or “always.” The subscale and total scores were converted to a range of 0-100 for easier interpretation, where higher scores indicate higher adherence.

We assessed food intake using dietary recall. It is a tool to estimate the consumption of meals throughout the day, including breakfast, breakfast snack, lunch, evening snack, dinner, and bedtime. It also takes into account the type of food, its components, and the amount consumed. It is conducted through interviews over a span of three days, with two days scheduled during the week and one day on the weekend. Analysis of dietary recall was conducted using Nutritionist IV (N4) nutritional software. The items included energy, carbohydrates, protein, iron, zinc, folate, vitamin C, cholesterol, fiber, saturated fat, MUFA and PUFA.

Physical activity was assessed using the International Physical Activity Questionnaire (IPAQ). In this study, the short form of the questionnaire was used. This short form was proposed as an international physical activity measurement tool in 1988 by the World Health Organization (WHO) and the Centers for Disease Control and Prevention (CDC) for the age group of 15-69 years. Its validity and reliability have been checked and confirmed in several studies. Also, a study conducted in Iran validated the Farsi version of this questionnaire [39]. Results can be reported in categories (low activity levels, moderate activity levels, or high activity levels) or as a continuous variable (MET minutes per week). MET minutes represent the amount of energy expended during physical activity.

Experienced researchers gathered the data based on the study protocol (Supporting File 3). Medical history taking as well as physical examinations (assessment of diastolic and systolic blood pressure [BP]) were done for determination of the obesity related comorbidities and assessment of the participants’ general health status. Baseline information, including demographic variables (gender and age), were gathered, and anthropometric indices (height, waist circumference, weight, and body mass index [BMI]) were assessed based on the World Health Organization (WHO) instructions [40]. Blood sampling was done before the surgery. Following 12–14 h of overnight fasting, blood specimens were collected and used to determine iron profile (serum iron level and ferritin), as well as hemoglobin (Hb) and hematocrit. Blood Hb and serum ferritin levels were measured using the cyanmethemoglobin method and human ferritin enzyme immunoassay test, respectively. Serum iron was determined using the spectrophotometric method.

Body composition was measured by a bioelectrical impedance analyzer (InBody 370, South Korea) at the baseline and 6-, 12-, 24- and 36-month postsurgery. We asked the subjects to obey some measures prior to impedance analysis as follows: fasting overnight or for at least 4–5 h, no physical activity for at least 12 h and no use of alcohol for at least 24 h before the test, maintaining balanced hydration and lying on your back for at least 5 min before test. Resistance against an alternating current (500 lA at 50/60 kHz) was assessed while standing on the analyzer’s platform and the results were analyzed by the software’s “standard” option. Fat mass (FM) and fat-free mass (FFM) were assessed. The BIA validity to detect body composition changes in cases subjected to bariatric surgery has been already confirmed [41,42].

The amount of weight loss was calculated as the excess weight loss percentage (EWL%) using the following formula: EWL% =  100 ×  (ideal weight - preoperative weight)/ (current weight - preoperative weight). The ideal weight for individuals with a BMI of 25 kg/m2 was considered the reference weight.

In this study, the comparison of difference change was based on the self-management behaviors using the Bariatric Surgery Self-Management Behaviors Questionnaire (BSSQ) score that Primary outcomes and secondary outcome included dietary recall, IPAQ, structured questionnaire based on HAPA, EWL and Blood chemistry.

### Intervention

The educational intervention was designed and implemented based on the constructs of the HAPA. These constructs include behavioral intention, task and coping self-efficacy, recovery self-efficacy, risk perception, outcome expectancies, action planning, and coping planning. The intervention group received a patient self-management approach intervention, which focused on promoting a healthy diet and physical activity over a period of two months. The intervention was conducted through group training and discussion sessions, distribution of pamphlets, and the sending motivational and persuasive messages. Weekly sessions lasting 45 to 60 minutes were held. The participants received three pamphlets containing educational content on diet, food basket, and physical activity, as well as a checklist of tasks for planning. Homework checklists were reviewed weekly, and feedback was provided to the individuals. In the first four weeks, they presented information on the structures of risk perception, outcome expectations, task and coping self-efficacy, and the intention to adopt appropriate dietary behaviors and engage in physical activity. During the second four weeks, we introduced action planning, coping planning, and recovery self-efficacy to promote a healthy diet and physical activity. Action and coping planning were presented in a series of consecutive meetings. The action planning included determining when, where, and how a behavior would be performed, identifying potential barriers, and developing coping strategies. Patients were asked to prepare a diet and physical activity plan, which should include information on when, where, and how to follow it. Participants were also asked to plan their behavior in the tempting situation. The following week, participants reported on the success rate of their action and coping planning. During the two-month intervention, participants were also contacted via telephone calls to provide feedback on their homework checklists and address any problems that they may have encountered. The calls encouraged self-efficacy and planning, identification of probable barriers, and development of coping strategies.

Educational and motivational text messages were also used to facilitate the implementation of action planning in patients. Patients in the control group received standard care at the center.

They received nutritional and exercise recommendations under the supervision of the center as part of their routine.

### Statistical analysis

The mean and standard deviation were used to describe the data, and a Paired t- test was used to compare the variables within groups. To compare the results between the two groups before the intervention, the independent t-test was used. Finally, to compare the changes between the two groups, we used generalized estimating equations (GEE) with unstructured working covariance structure using robust estimation method as this model do not need normal distribution assumption for the outcome and could handle the any correlation structure. In this model the effecmain effect time and interaction of the time and group were included into this model (We perfectly remove the main effect of the group to adjust for the baseline). Although all these terms included, the main outcome (the difference of changes in the responses) would be inferenced from the interaction of the time and group (other parts provide different information) [43].To adjust for multiple comparisons, we used the approach of Benjamin & Hochberg [44]. All statistical analysis was performed using SPSS (version 23.0) and R. Statistical significance was considered for p-value of less than 0.05.

## Results

Most of the participants were female (86% of the intervention and 74% of the control group). Regarding age of the participants, 47.9% of the intervention group and 48.9% of the control group were 36–50 years old. No significant differences were observed between the two study groups for the demographic variables at baseline (Table 1). The mean iron level in the intervention group significantly increased compared to the control group after the educational intervention (P = 0.03) in the inter-group comparison adjusted for the basline. In the intra-group comparison, the hemoglobin score in the intervention group increased significantly following the intervention compared to the baseline (p = 0.01). It also increased in the control group, but the change was not statistically significant (p =  0.07) (Table 2). According to Table 3, in the inter-group comparison adjusted for the basline, the scores of the action and coping self-efficacy (P = 0.04) constructs, action planning (P < 0.01), and behavioral intention (P = 0.02) in the intervention group increased significantly compared to the control group after the educational intervention.. In the intragroup comparison, a significant increase was observed in the action and coping self-efficacy in the intervention group compared to the baseline (p <  0.02). The score in the control group did not increase significantly (p =  0.76). The action planning score in the intervention group showed a significant increase compared to the baseline (p =  0.02), while in the control group, a decrease of 0.26 was observed, which was not significant (p =  0.12). In the inter-group comparison adjusted for the basline, the scores of the action planning (P = 0.02), risk perception (P = 0.01), and outcome expectations (P = 0.01) significantly increased in the intervention group compared to the control group after the educational intervention.

**Table 1.** Demographic characteristics of the study participants in both intervention and control groups at baseline (n =  100).

Variable	Level	Total	Intervention	Group	Control	P
						0.13
Gender	Female	80(80%)	43(86%)		37(74%)	
	Male	20(20%)	7(14%)		13(26%)	
Age	18-35	37(38.9%)	18(37.5%)		19(40.4%)	0.84
	36-50	46(48.4%)	23(47.9%)		23(48.9%)	
	50 <	12 (12.6%)	7(14.6%)		5(10.6%)	
Education	Illiterate	2(2.0%)	2(4%)		0(0%)	0.35
	Diploma	42(42%)	23(46%)		19(38%)	
	Bachelor’s 34(34%) Degree		16(32%)		18(36%)	
	Postgraduate 22(22%)		9(18%)		13(26%)	
Marital status	Single	28(28%)	15(30%)		13(26%)	0.65
	Married	72(72%)	35(70%)		37(74%)	
Economics	Weak	4(4%)	2(4%)		2(4%)	0.25
	Moderate	80(80%)	37(74%)		43(86%)	
	Good	16(16%)	11(22%)		5(10%)	

**Table 2.** Blood chemical parameters and BMI, IPAC in the intervention and control groups.

Parameter	Time	Group		Diff	95% CI		P†	P for post adjusted for baseline
		Control	Intervention		Lower	Upper		
Hb	Baseline	13.04 ± 1.81	13.03 ± 1.52	0.13	-0.53	0.81	**0.67** ^**c**^	
	Post	13.06 ± 1.82	13.06 ± 1.53	-0.02	-0.74	0.68		
	Change	-0.15 ± 0.51	-0.25 ± 0.66	-0.10	-0.16	0.36		**0.71** ^**e**^
	P-within‡	**0.07** ^**a**^	**0.01** ^**a**^					
Hct	Baseline	40.72 ± 4.31	39.61 ± 4.52	1.03	-0.79	2.86	**0.26** ^**c**^	
	Post	40.74 ± 4.31	40.32 ± 4.71	0.41	-0.1.51	2.34		
	Change	-0.11 ± 1.43	-0.58 ± 2.12	0.46	-0.29	1.22		**0.36** ^**e**^
	P-within‡	**0.58** ^**a**^	**0.07** ^**a**^					
Fe	Baseline	80.41 ± 30.12	73.62 ± 27.71	6.84	-4.95	18.61	**0.29** ^**d**^	
	Post	77.11 ± 29.04	79.06 ± 31.21	-1.94	-14.61	10.72		
	Change	1.72 ± 10.81	-5.34 ± 16.21	7.06	1.26	12.81		**0.03** ^**e**^
	P-within‡	**0.61** ^**b**^	**0.01** ^**b**^					
BMI	Baseline	28.31 ± 4.71	28.72 ± 5.31	-0.42	-2.41	1.51	**0.71** ^**d**^	
	Post	27.91 ± 4.52	27.71 ± 4.22	0.19	-1.61	1.99		
	Change	0.35 ± 1.31	0.61 ± 2.27	-0.27	-1.02	0.47		**0.98** ^**e**^
	P-within‡	**0.09** ^**b**^	**0.04** ^**b**^					
IPAC	Baseline	1205.21 ± 1633.71	1103.88 ± 1290.34	101.32	-513.91	716.51	**0.86** ^**d**^	
	Post	1138.36. ± 2117.84	2359.54 ± 3553.39	-1221.11	-2482.21	39.85		
	Change	-26.92 ± 2116.21	-1038.15 ± 3190.05	1011.21	-231.51	2254.04		**0.81** ^**e**^
	P-within‡	**0.89** ^**b**^	**0.05** ^**b**^					

^a^paired t-test, ^b^Wilcoxon signed-rank test, ^c^Independent Samples T-Test, ^d^Mann–Whitney, ^e^ Adjusted for baseline based on interaction of time and group within a GEE analysis *α<0.05

Some discrepancy between the change and mean of pre and post were due to missing values for some variables.

**Table 3.** Healthy diet questionnaire based on the constructs of HAPA among Bariatric surgery patients.

Parameter	Time	Group		Diff	95% CI		P†	P for post adjusted for baseline
		Control	Intervention		Lower	Upper		
Task and Coping	Baseline	3.44 ± 1.04	3.45 ± 0.96	-0.01	-0.41	0.38	**0.94** ^**c**^	
Self-Efficacy	Post	3.48 ± 0.99	3.92 ± 0.61	-0.43	-0.78	-0.09		
	Change	-0.46 ± 1.11	-0.42 ± 0.95	0.38	-0.03	0.80		**0.02** ^**e**^
	P-within‡	**0.76** ^**a**^	**0.01** ^**a**^					
Coping planning	Baseline	3.58 ± 1.08	3.82 ± 0.93	-0.24	-0.63	0.14	**0.19** ^**d**^	
	Post	3.39 ± 0.97	3.94 ± 0.68	-0.55	-0.89	0.20		
	Change	0.19 ± 1.09	-0.13 ± 0.98	0.32	-0.08	0.73		**0.01** ^**e**^
	P-within‡	**0.08** ^**b**^	**0.66** ^**b**^					
Action planning	Baseline	3.78 ± 1.07	4.08 ± 0.77	-0.31	-0.67	0.07	**0.24** ^**d**^	
	Post	3.52 ± 1.13	4.36 ± 0.49	-0.83	-1.92	-0.47		
	Change	0.26 ± 1.09	-0.31 ± .0.92	0.57	0.16	0.99		**0.01** ^**e**^
	P-within‡	**0.15** ^**b**^	**0.02** ^**b**^					
Risk perception	Baseline	4.06 ± 0.84	4.21 ± 0.87	-0.15	-0.49	0.18	**0.28** ^**d**^	
	Post	4.25 ± 0.80	4.75 ± 0.35	-0.51	-0.75	-0.24		
	Change	-0.19 ± 0.83	-0.52 ± 0.91	0. 32	-0.02	0.68		**0.41** ^**e**^
	P-within‡	**0.17** ^**b**^	**0.01** ^**b**^					
Outcome	Baseline	4.66 ± 0.63	4.71 ± 0.64	-0.04	-0.29	0.21	**0.81** ^**d**^	
Expectancies	Post	4.61 ± 0.71	4.95 ± 0.11	-0.33	-0.54	-0.12		
	Change	0.45 ± 0.51	-0.26 ± 0.65	0.30	0.06	0.54		**0.06** ^**e**^
	P-within‡	**0.40** ^**b**^	**0.01** ^**b**^					
Recovery self-	Baseline	3.56 ± 1.26	3.93 ± 1.05	-0.36	-0.82	0.09	**0.13** ^**d**^	
Efficacy								
	Post	3.86 ± 1.19	4.60 ± 0.61	-0.74	-1.13	-0.34		**0.07** ^**e**^
	Change	-0.30 ± 1.19	-0.63 ± 1.07	0.33	-0.15	0.83		
	P-within‡	**0.25** ^**b**^	**0.01** ^**b**^					
Intention	Baseline	4.03 ± 1.08	4.06 ± 1.04	-0.03	-0.45	0.39	**0.95** ^**d**^	
	Post	3.81 ± 1.16	4.54 ± 0.53	-0.74	-1.11	-0.36		
	Change	0.23 ± 1.20	-0.46 ± 1.15	0.69	0.21	1.17		**0.01** ^**e**^
	P-within‡	**0.18** ^**b**^	**0.01** ^**b**^					

^a^paired t-test, ^b^Wilcoxon signed-rank test, ^c^Independent Samples T-Test, ^d^Mann–Whitney, ^e^ Adjusted for baseline based on interaction of time and group within a GEE analysis *α<0.05

Some discrepancy between the change and mean of pre and post were due to missing values for some variables.

In the intra-group comparison of the intervention and control groups, the scores of the action and coping self-efficacy in the intervention group significantly increased compared to the baseline (p = 0.01). No significant increase was found in the control group (p =  0.98). The action planning score in the intervention group showed a significant increase compared to the baseline (p =  0.01). In the control group, there was no significant increase (p =  0.63) (Table 4). According to Table 5, in the inter-group comparison adjusted for the basline, the scores for physical activity (P < 0.01) in the intervention group significantly increased compared to the control group after the educational intervention. In the inter-group comparison adjusted for the basline, the dietary recall score in the intervention group did not show significant increase after the educational intervention, as compared to the control group (Table 6). No significant EWL was found (P =  0.71, Diff =  2.22, 95% CI: -3.59 to 5.22).

**Table 4.** Physical activity questionnaire based on the constructs of HAPA among Bariatric surgery patients.

Parameter	Time	Group		Diff	95% CI		P†	P for post adjusted for baseline
		Control	Intervention		Lower	Upper		
	Baseline	3.88 ± 1.15	3.94 ± 1.16	-0.06	-0.52	0.40	**0.68** ^**d**^	
Task and coping self-efficacy								
	Post	3.89 ± 1.08	4.37 ± 0.92	-0.47	-0.89	0.06		
	Change	-0.27 ± 1.04	-0.46 ± 1.15	0.43	-0.01	0.89		**0.76** ^**e**^
	P-within‡	**0.98** ^**b**^	**0.01** ^**b**^					
Action planning	Baseline	3.86 ± 0.84	3.94 ± 1.03	-0.08	-0.45	0.29	**0.33** ^**d**^	
	Post	3.86 ± 0.98	4.26 ± 0.76	-0.39	-0.76	-0.03		
	Change	-0.02 ± 0.76	-0.4 ± 0.84	0.38	0.05	0.70		**0.02** ^**e**^
	P-within‡	**0.63** ^**b**^	**0.01** ^**b**^					
Coping planning	Baseline	3.91 ± 0.98	3.94 ± 0.89	-0.03	-0.41	0.33	**0.91** ^**d**^	
	Post	3.82 ± 1.05	4.04 ± 0.88	-0.22	-0.62	0.17		
	Change	0.081 ± 0.88	-0.11 ± 0.68	0.21	-0.12	0.52		**0.37** ^**e**^
	P-within‡	**0.51** ^**b**^	**0.29** ^**b**^					
Risk perception	Baseline	4.21 ± 0.95	4.24 ± 0.98	-0.02	-0.41	0.35	**0.74** ^**d**^	
	Post	4.09 ± 0.95	4.63 ± 0.59	-0.54	-0.87	-0.21		
	Change	0.10 ± 1.13	-0.41 ± 0.88	0.51	0.08	0.92		**0.01** ^**e**^
	P-within‡	**0.50** ^**b**^	**0.01** ^**b**^					
Outcome Expectancies	Baseline	4.79 ± 0.56	4.73 ± 0.58	0.06	-0.16	0.28	**0.14** ^**d**^	
	Post	4.63 ± 0.69	4.83 ± 0.32	-0.20	-0.42	0.02		
	Change	0.15 ± 0.57	0.03 ± 0.60	0.25	0.01	0.50		**0.06** ^**e**^
	P-within‡	**0.04** ^**b**^	**0.44** ^**b**^					
Recovery self- efficacy	Baseline	3.82 ± 1.43	4.15 ± 1.02	0.32	-0.82	0.16	**0.53** ^**d**^	
	Post	3.98 ± 1.08	4.38 ± 0.80	-0.39	-0.79	-0.03		**0.01** ^**e**^
	Change	-0.18 ± 1.20	-0.27 ± 1.07	0.09	0.38	0.56		
	P-within‡	**0.66** ^**b**^	**0.12** ^**b**^					
Intention	Baseline	4.03 ± 1.01	4.19 ± 0.94	-0.16	-0.55	0.23	**0.37** ^**d**^	
	Post	4.00 ± 1.11	4.35 ± 0.71	-0.35	-0.74	0.03		
	Change	0.01 ± 1.05	-0.20 ± 0.84	0.21	-0.18	0.60		**0.15** ^**e**^
	P-within‡	**0.95** ^**b**^	**0.16** ^**b**^					

^a^paired t-test, ^b^Wilcoxon signed-rank test, ^c^Independent Samples T-Test, ^d^Mann–Whitney, ^e^Adjusted for baseline based on interaction of time and group within a GEE analysis *α<0.05

Some discrepancy between the change and mean of pre and post were due to missing values for some variables.

**Table 5.** BSSQ questionnaire based on the constructs of HAPA among Bariatric surgery patients.

Parameter	Time	Group		Diff	95% CI		P†	P for post adjusted for baseline
		Control	Intervention		Lower	Upper		
Eating behaviors	Baseline	53.81 ± 0.16	59.01 ± 16.51	-5.12	-11.06	1.39	**0.12** ^**c**^	
	Post	58.21 ± 17.61	73.01 ± 14.12	-14.07	-21.31	-8.11		
	Change	-4.51 ± 19.71	-13.71 ± 19.21	9.11	1.12	17.09		**0.25** ^**e**^
	P-within‡	**0.11** ^**a**^	**0.01** ^**a**^					
Fruit intake	Baseline	53.61 ± 29.91	54.31 ± 29.82	-0.66	-12.54	11.21	**0.81** ^**d**^	
	Post	56.81 ± 23.05	66.48 ± 25.72	-9.67	-19.68	0.32		
	Change	-2.72 ± 24.11	-10.55 ± 30.41	7.83	-3.37	19.04		**0.78** ^**e**^
	P-within‡	**0.68** ^**b**^	**0.05** ^**b**^					
Protein intake	Baseline	56.00 ± 29.31	57.06 ± 28.06	-1.66	-13.01	9.82	**0.95** ^**d**^	
	Post	60.21 ± 20.91	69.92 ± 21.04	-9.72	-18.04	1.02		
	Change	-4.08 ± 27.05	-10.06 ± 29.03	-6.58	-5.07	18.24		**0.95** ^**e**^
	P-within‡	**0.49** ^**b**^	**0.01** ^**b**^					
Physical activity	Baseline	55.03 ± 30.01	50.01 ± 31.05	5.33	-6.89	17.56	**0.34** ^**d**^	
	Post	49.03 ± 26.03	61.53 ± 30.09	-12.21	-23.09	-0.47		
	Change	5.01 ± 26.06	-12.06 ± 34.09	17.07	5.08	30.41		**0.83** ^**e**^
	P-within‡	**0.12** ^**b**^	**0.02** ^**b**^					
Dumping syndrome	Baseline	51.01 ± 32.05	53.01 ± 28.05	-2.01	-14.14	10.14	**0.83** ^**d**^	
	Post	46.02 ± 30.8	51.01 ± 240.9	-4.85	-16.4	6.71		
	Change	5.78 ± 29.05	1.11 ± 21.04	4.67	-5.99	15.03		**0.73** ^**e**^
	P-within‡	**0.25** ^**b**^	**0.84** ^**b**^					
Fluid intake	Baseline	54.51±2.07	570.3±21.01	-2.87	-11.01	5.44	**0.76** ^**d**^	
	Post	58.21 ± 18.02	60.81 ± 18.09	-2.54	-10.11	4.91		
	Change	-3.57 ± 17.03	-1.94 ± 15.81	-1.62	-8.43	5.18		**0.98** ^**e**^
	P-within‡	**0.18** ^**b**^	**0.48** ^**b**^					
Mineral intake	Baseline	54.25 ± 26.19	53.25 ± 24.45	1.01	-9.05	11.05	**0.92** ^**d**^	
	Post	50.25 ± 24.93	58.0 8 ± 26.31	-8.54	-19.04	1.95		
	Change	4.08 ± 24.12	-7.41 ± 22.03	11.49	1.94	21.04		**0.97** ^**e**^
	P-within‡	**0.28** ^**b**^	**0.02** ^**b**^					

^a^paired t-test, ^b^Wilcoxon signed-rank test, ^c^Independent Samples T-Test, ^d^Mann–Whitney, ^e^ Adjusted for baseline based on interaction of time and group within a GEE analysis, *α<0.05.

Some discrepancy between the change and mean of pre and post were due to missing values for some variables.

**Table 6.** Dietary recall based on the constructs of HAPA among Bariatric surgery patients.

Parameter	Time	Group		Diff	95% CI		P†	P for post adjusted for baseline
		Control	Intervention		Lower	Upper		
Energy	Baseline	1604.61 ± 492.41	1805.71 ± 626.61	-201.11	-424.71	22.05	**0.09** ^**d**^	
	Post	1712.09 ± 559.05	1855.7 ± 578.4	-143.61	-375.51	88.02		
	Change	-107.04 ± 608.4	-106.4 ± 543.3	-1.03	-237.11	235.04		**0.15** ^**e**^
	P-within‡	**0.29** ^**b**^	**0.11** ^**b**^					
	Baseline	206.41 ± 81.41	213.12 ± 93.31	-6.68	-39.04	26.02	**0.68** ^**c**^	
Carbohydrate	Post	212.71 ± 60.81	214.71 ± 75.11	-2.01	-29.07	25.07		
	Change	-6.27 ± 76.41	0.10 ± 63.61	-6.38	-35.02	22.04		**0.91** ^**e**^
	P-within‡	**0.56** ^**a**^	**0.99** ^**a**^					
	Baseline	18.04 ± 7.03	21.04 ± 11.02	-2.64	-6.04	1.15	**0.43** ^**d**^	
MUFA	Post	20.09 ± 11.01	21.05 ± 8.03	-0.55	-4.05	3.47		
	Change	-2.05 ± 13.09	-0.62 ± 12.07	-1.95	-7.01	3.01		**0.38** ^**e**^
	P-within‡	**0.42** ^**b**^	**0.31** ^**b**^					
Protein	Baseline	66.31 ± 21.43	75.05 ± 32.06	-9.23	-20.18	1.72	**0.22** ^**d**^	
	Post	66.52 ± 19.73	83.61 ± 22.66	-17.09	-25.73	-8.45		
	Change	-0.21 ± 24.02	-8.42 ± 33.00	8.02	-3.05	19.92		**0.01** ^**e**^
	P-within‡	**0.58** ^**b**^	**0.01** ^**b**^					
Saturated fat	Baseline	18.31 ± 6.04	20.81 ± 9.01	-2.46	-5.06	0.67	**0.12** ^**c**^	
	Post	21.61 ± 9.06	20.41 ± 7.07	1.26	-2.32	4.85		
	Change	-3.03 ± 11.06	0.44 ± 11.08	-3.77	-8.57	1.01		**0.21** ^**e**^
	P-within‡	**0.04** ^**a**^	**0.80** ^**a**^					
Zinc	Baseline	7.03 ± 2.05	8.07 ± 6.06	-1.41	-3.25	-0.43	**0.54** ^**d**^	
	Post	8.05 ± 4.08	9.23 ± 3.91	-0.72	-2.05	1.09		
	Change	-1.15 ± 4.08	-0.60 ± 5.07	-0.55	-2.73	1.61		**0.28** ^**e**^
	P-within‡	**0.22** ^**b**^	**0.08** ^**b**^					
PUFA	Baseline	12.08 ± 7.04	14.09 ± 12.08	-1.25	-5.42	2.90	**0.95** ^**d**^	
	Post	13.07 ± 12.02	14.09 ± 9.39	-1.27	-5.76	3.21		
	Change	-0.86 ± 14.06	-0.95 ± 14.01	0.08	-5.79	5.97		**0.72** ^**e**^
	P-within‡	**0.98** ^**b**^	**0.16** ^**b**^					
Vitamin C	Baseline	85.03 ± 69.01	77.07 ± 68.08	7.54	-19.85	34.93	**0.66** ^**d**^	
	Post	120.01 ± 72.04	130.09 ± 90.02	-10.76	-43.95	22.42		
	Change	-34.08 ± 89.01	-50.05 ± 101.09	15.71	-23.21	54.65		**0.65** ^**e**^
	P-within‡	**0.07** ^**b**^	**0.01** ^**b**^					
Cholesterol	Baseline	306.07 ± 196.01	319.06 ± 212.04	-12.09	-94.08	68.20	**0.81** ^**d**^	
	Post	324.04 ± 195.03	335.01 ± 168.02	-10.06	-85.28	64.08		
	Change	-17.06 ± 232.01	-10.2 ± 269.06	-7.04	-109.05	94.06		**0.92** ^**e**^
	P-within‡	**0.92** ^**b**^	**0.64** ^**b**^					
Folate	Baseline	228.03 ± 127.03	232.03 ± 111.08	-4.02	-51.05	43.05	**0.47** ^**d**^	
	Post	242.03 ± 122.01	255.01 ± 104.08	-12.07	-59.02	33.07		
	Change	-14.05 ± 164.04	-17.09 ± 135.07	3.89	-57.09	65.07		**0.87** ^**e**^
	P-within‡	**0.31** ^**b**^	**0.34** ^**b**^					
Iron	Baseline	12.81 ± 5.81	13.51 ± 6.21	-0.69	-3.08	1.69	**0.40** ^**d**^	
	Post	12.03 ± 6.01	13.81 ± 4.51	-1.57	-3.07	0.63		
	Change	0.56 ± 7.61	-0.52 ± 5.59	1.08	-1.06	3.83		**0.31** ^**e**^
	P-within‡	**0.34** ^**b**^	**0.02** ^**b**^					
Dietary fiber	Baseline	12.08 ± 6.09	11.09 ± 6.23	0.88	-1.56	3.32	**0.71** ^**d**^	
	Post	13.04 ± 7.35	14.08 ± 5.25	-0.66	-3.29	1.96		
	Change	-0.58 ± 7.92	-1.86 ± 6.91	1.28	-1.76	4.33		**0.61** ^**e**^
	P-within‡	**0.50** ^**b**^	**0.01** ^**b**^					

^a^paired t-test, ^b^Wilcoxon signed-rank test, ^c^Independent Samples T-Test, ^d^Mann–Whitney, ^e^ Adjusted for baseline based on interaction of time and group within a GEE analysis, *α<0.05

Some discrepancy between the change and mean of pre and post were due to missing values for some variables.

## Discussion

In this randomized controlled trial, our aim was to assess an intervention developed using the Health Action Process Approach to enhance the outcomes of bariatric surgery in patients with obesity. Our findings indicated that HAPA intervention could serve as an effective adjunct to patients’ treatment.

Individuals who have undergone bariatric surgery often experience weight regain and demonstrate a lack of engagement in self-care and self-management activities following the procedure [19,45–47].Effective self-management following bariatric surgery is essential for achieving successful weight loss and sustaining weight reduction [48,49].

At the baseline measurement, there were no significant differences in demographic characteristics between the intervention and control groups, indicating that the results of this study were minimally affected by confounding factors.

The predominant demographic in our study comprised female individuals, accounting for 80% of the participants, with the average age falling within the 36 to 50 range for both groups. Young et

al. reported a similar gender distribution, with 80.7% of their study’s participants being female. Nevertheless, Young’s study findings indicated a rising prevalence of bariatric surgery among male patients over a 9-year timeframe [50].

Our study demonstrated the effectiveness of the educational intervention in participants. Generally, patient activation interventions utilizing the HAPA had a positive impact on self-management of healthy diet and physical activity among bariatric surgery patients. It is important to note the scarcity of studies specifically focusing on the application of the HAPA model in this patient population. Nevertheless, research studies such as Ranjbaran et al.’s investigation on diabetes has indicated the effectiveness of this approach in promoting dietary adherence [34]. Additionally, Lin et al.’s study in Iran reported an increase in fruit and vegetable consumption among adolescents following the HAPA-based intervention [51]. Furthermore, findings from other studies have indicated that the HAPA framework can be a suitable and practical approach for intervention plans aimed at promoting physical activity in adults with obesity [52–55].

Action self-efficacy is considered a significant determinant in the intention, action planning, and coping planning within the context of the health behavior process model. These determinants collectively reduce the gap between intention and actual behavior [56]. Furthermore, a lack of self-efficacy is a contributing factor in hindering the educational process for patients, leading to social withdrawal and diminished educational expectations [57]. Following our intervention, the intervention group exhibited a significant increase in self-efficacy action and coping scores compared to the control group. Walker et al. found that high self-efficacy was linked to enhanced self-care behaviors, such as healthy eating and diet, in patients [58]. Similarly, Batsis et al. demonstrated that obese adults who underwent bariatric surgery and achieved sustained weight loss experienced an enhancement in self-efficacy, which is consistent with the findings of our study [59].

Following the implementation of the educational program, it was noted that the intervention group exhibited an augmentation in their self-management intention concerning their dietary habits. This enhancement was linked to the delivery of lectures, interactive question and answer sessions, distribution of educational pamphlets, and dissemination of educational short message texts. These materials were designed based on the needs assessment of the baseline results and aimed to enhance patients’ intention to manage their diet effectively. It is evident that the intention to manage diet improved more significantly than physical activity, potentially due to the fact that patients had a higher score in diet self-management at the outset of the study. Research conducted by Lin et al and Bierbauer et al demonstrated that intention increased subsequent to an educational intervention aimed at enhancing dietary habits and adherence to medication regimens [51,60]. Following the formation of an intention, individuals with high self-efficacy are better equipped to initiate and sustain behavioral changes with less difficulty compared to those with low self-efficacy [60]. These findings align with the outcomes of our study.

Following the intervention, the intervention group demonstrated an improvement in their performance planning score in relation to diet and physical activity. Given the multifaceted nature of behavior, interventions aimed at behavior modification should take into account additional factors, such as action planning and coping strategies. Individuals should equip themselves with preparatory strategies to facilitate the execution of their intentions. Planning is a crucial strategy that influences the certainty of intention [61]. To enhance planning effectiveness, individuals may benefit from seeking guidance from experienced professionals, engaging in daily planning, and drawing from personal experiences to bolster self-efficacy [62]. Planning interventions can be effective in promoting more frequent action planning among participants [63,64]. The lack of a significant increase in coping planning score in this study is likely attributed to the presence of various obstacles hindering behavior performance in this group. Future studies should aim to comprehensively identify these obstacles and gain a deeper understanding of these barriers and the role of coping planning in patients following bariatric surgery using appropriate methodologies. The findings of Ghisi et al.‘s study, utilizing the HAPA on cardiac rehabilitation patients, revealed a notable increase in action and coping planning in the context of physical activity post-intervention [65]. Scholz et al.’s study results demonstrated that the alteration in action planning, particularly performance control, is pivotal in modifying two distinct behaviors: smoking cessation and nutritional behavior in individuals adhering to a long-term diet [66].

The study also revealed that there was no significant increase in the recovery self-efficacy scores related to diet self-management following the intervention in the experimental group. Bierbauer et al. demonstrated in their research that older individuals with a strong sense of self-efficacy in resuming their medication regimen showed improved adherence to prescribed medications, indicating the importance of self-efficacy in recovery as a predictor of adherence to behavior [60]. This finding contrasts with the results of the current study. The inconsistency in the findings may be attributed to the duration of follow-up for the target group. It is essential to conduct follow-ups at longer intervals than those mentioned in order to assess the structure of recovery self-efficacy. This is because individuals need to first implement health recommendations and may experience relapse to previous behaviors and habits during program implementation. Ultimately, they rely on recovery self-efficacy to return to healthy behavior after a relapse, gradually regaining their intended behavioral performance [67]. The primary application of self-efficacy is to aid individuals in resuming their actions when they have been interrupted or stopped [67]. In this study, relapse prevention was implemented by assisting patients in evaluating potential high-risk situations, analyzing their past responses to stimuli, and introducing new strategies to address future obstacles and challenges. The variation in self-efficacy among individuals results in the implementation of different strategies based on the patient’s condition. Patients have the confidence that even if they have not followed their diet or engaged in physical activity due to unfortunate circumstances, they can still start over and return to the target behavior cycle. Another factor that diminishes the significance of this construct is the lack of support from family and friends. When patients eat with family members and friends, they often do not choose healthy foods, leading to a disregard of nutritional guidelines and a return to old behaviors.

The results of the current study demonstrate that the framework of outcome expectations no significantly influences the intention to engage in physical activity behaviors. Following the intervention, there was a notable increase in individual scores, underscoring the importance of considering this concept in lifestyle interventions. Additionally, perceived risk serves as a crucial motivational factor for embracing health behaviors, particularly among post-surgical patients compared to individuals in good health. The former group encounters more frequent health threats in their daily lives and is thus more inclined to prioritize their well-being. In a study by Jihouni et al., it was observed that the implementation of an educational intervention aimed at discouraging hookah use led to a substantial increase in both risk perception and outcome expectations. These findings align with the results of the present study [68].

In our study, the serum iron level score exhibited a significant increase in the intervention group compared to the control group following the intervention. Research has demonstrated deficiencies in iron, ferritin, vitamins, and minerals subsequent to bariatric surgery, underscoring the significance of equipping patients with knowledge, awareness, and educational interventions to adhere to a nutritious diet [10,69–73]. Alshwaiyat et al.‘s study revealed a noteworthy enhancement in weight loss among obese female participants who adhered to a healthy diet. Additionally, the intervention resulted in an amelioration of the patients’ iron status, which is consistent with the findings of our current study [74]. Furthermore, post-surgery, patients often consume less food and have a reduced intake of iron, particularly from meat [75]. Therefore, requesting pertinent tests in this context and interpreting their outcomes can effectively contribute to the identification and management of iron deficiency and other hematological parameters subsequent to bariatric surgery.

In our study, the food intake score did not show significant increase in the intervention group after the intervention. Food estimation was measured using the dietary recall method, which relies on the memory of the participants, and there is a possibility of recall bias.

### Strengths and Limitations of the Study

In this study, we recorded weekly action plans and coping plans through collecting planning checklists, rather than relying on self-reporting for planning. Also, there were no drop-out rate among the participants during the intervention. Some limitations need to be addressed in this study. Data collection was self-reported. However, the researchers made efforts to gain the trust of the patients by emphasizing the confidentiality of the data.

### Implications for practice

The findings of this study can assist physicians and healthcare providers in developing effective programs to enhance postoperative outcomes for bariatric surgery patients and address issues related to non-adherence and self-management. Educational interventions in this field are both appropriate and cost-effective. By improving self-management skills, the likelihood of obesity recurrence and the necessity for repeat surgeries can be significantly reduced. Support from family and friends, as well as participation in educational interventions, is crucial for achieving better outcomes. Future research should consider longer follow-up periods to evaluate dietary compliance and physical fitness over an extended timeframe. Increasing the duration of the intervention may enhance its effectiveness and improve patient adherence. Additionally, this study utilized virtual interventions; conducting training sessions in person may yield more favorable results.

## Conclusion

Our study demonstrated the effectiveness of an intervention designed using the Health Action Process Approach in promoting proper diet and regular physical activity among patients one year after bariatric surgery. A combination of group therapy, online interventions, and reminder strategies resulted in higher blood serum iron levels and improved eating habits in patients four months after the intervention. This approach can be utilized as a suitable method to complement the treatment of patients.

## Supporting information

S1 File CONSORT 2010 checklist. (DOC)

S2 File The Questionnaire. (DOCX)

S3 File Data. (XLSX)
