# Supplementary material for: Effectiveness of an intervention designed based on the Health Action Process Approach on obesity surgery outcomes in patients who have undergone bariatric surgery after one year: A randomized controlled trial
Source: PLoS One. 2025 Apr 9;20(4):e0314316. doi: 10.1371/journal.pone.0314316 (PMC11981231; doi:10.1371/journal.pone.0314316)
Supplement: S2 File — (DOCX) [file pone.0314316.s002.docx]

| **Healthy Diet Items** | |
| --- | --- |
| Questions | Construct |
| I can follow the diet recommended by my doctor, even if: | Task Self-Efficacy and Coping |
| I am traveling. |  |
| my friends don't help me to follow my diet. |  |
| I have a strong temptation to consume foods that are not recommended. |  |
| I am confident that if I don't follow my diet for a short period of time, I can get back on my diet again, even if: | Recovery Self-Efficacy |
| something bad has happened and I didn't follow my diet. |  |
| I was encouraged to eat unhealthy food for a while by my friends and family. |  |
| I have given up on my diet due to being away from my place of residence or traveling. |  |
| I have a detailed plan to follow my diet in the following cases: | Action Planning |
| What food I should eat. |  |
| When to start following my diet. |  |
| How to follow my diet. |  |
| After following my diet, I have detailed plan to: | Coping Planning |
| keep my workout schedule on track. |  |
| use reminders to help me follow my diet. |  |
| reject foods offered to me, which are outside of my diet plan. |  |
| avoid being around people who eat sweets, sugary foods, or fast food. |  |
| I know that if I don't follow the dietary recommendations given by my doctor: | Risk Perception |
| I will be upset with myself or I will doubt my willpower. |  |
| I will suffer from malnutrition. |  |
| the surgery’s side effects (such as hair loss, abdominal pain, fatigue, weakness, etc.) will increase. |  |
| my weight loss will slow down or will stop. |  |
| I know that if I follow my diet: | Outcome Expectations |
| I will have a better body image. |  |
| I will be more confident. |  |
| I will become refreshed and energetic enough to lead a better life. |  |
| I will reach my healthy weight. |  |
| I intend to follow my doctor’s instructions on my diet in the next six months | Behavioral Intentions |
| I plan to consume or avoid certain foods based on my diet in the next year |  |

| **Physical Activity Items** | |
| --- | --- |
| Items | Construct |
| I can: | Task Self-Efficacy and Coping |
| workout at least 30 minutes a day for five times a week. |  |
| lead a physically active lifestyle even if it's challenging for me. |  |
| workout even if there is no one to accompany me. |  |
| I am confident that I can resume my physical activity even if: | Recovery Self-Efficacy |
| I have given up working out several times. |  |
| I get sick. |  |
| people around me don't encourage me anymore. |  |
| For the next time, I have plans in detail about: | Action Planning |
| what physical activities I will do. |  |
| the weekdays I will work out on. |  |
| how long I will be physically active. |  |
| where to work out |  |
| For the next time, I have detailed plans to: | Coping Planning |
| cope with difficult situations in order to stick to my workout schedule. |  |
| use reminders to use to work out. |  |
| ways to keep my work out schedule on track. |  |
| Without physical activity: | Risk Perception |
| I will gain back my previous weight. |  |
| obesity-related issues will show up again. |  |
| my body skin will loosen. |  |
| If I exercise regularly: | Outcome Expectations |
| will maintain a healthy weight. |  |
| I will have a better image of myself. |  |
| I will feel refreshed. |  |
| I will be healthier |  |
| I plan: | Behavioral Intentions |
| to exercise for 20 minutes or more a day, at least 3 days a week |  |
| to exercise 30 minutes or more a day, at least 5 days a week |  |
